# The Organic Halo Effect: Perceived Caloric Disparities in High‐ and Low‐Calorie Foods and the Role of Nutrition Label Reading Frequency

**DOI:** 10.1111/jhn.70052

**Published:** 2025-04-21

**Authors:** Théo Besson, François Durand, Oulmann Zerhouni

**Affiliations:** ^1^ Laboratoire de psychologie sociale Université Paris Cité Paris France; ^2^ Laboratoire de Psychologie: Dynamiques Relationnelles Et Processus Identitaires (Psy‐DREPI – EA7458) Université de Bourgogne Franche‐Comté, Maison de l'Université Dijon France; ^3^ Laboratoire Parisien de Psychologie Sociale Université Paris Nanterre Nanterre France; ^4^ Centre de Recherche sur les Fonctionnements et Dysfonctionnements Psychologiques Université de Rouen Normandie Mont‐Saint‐Aignan France

**Keywords:** caloric perception, consumer behaviour, consumption recommendations, nutritional labelling, organic halo effect

## Abstract

**Background:**

This study examines the organic halo effect, specifically how organic labels influence perceptions of caloric content and consumption recommendations for high and low calorie food items. Previous research suggests that organic labels can create a perception of healthiness, but it is unclear how these perceptions vary with food calorie content.

**Methods:**

An online experiment was conducted with 198 participants, who were randomly assigned to evaluate 20 food items (10 high‐calorie and 10 low‐calorie) labelled as either organic or conventional. Participants rated the calorie content and recommended consumption frequency of each item using Likert scales. The analysis included multilevel regression models to account for nested data and interactions between labels, calorie content, and participants' propensity to read nutrition information.

**Results:**

The organic label led to a significant underestimation of calorie content for high‐calorie items and an overestimation for low‐calorie items. Participants who frequently read nutritional information were more sensitive to the organic label, showing a stronger organic halo effect. Consumption frequency recommendations were influenced by the label only for low‐calorie items, with conventional items being recommended more frequently.

**Discussion:**

The study shows that the organic halo effect is more pronounced for high‐calorie foods, leading to an underestimation of their calorie content, which may result in occasional overconsumption. Frequent readers of nutritional information are more susceptible to the organic halo effect, contradicting previous findings. The dissociation between calorie perception and consumption frequency highlights the need for clearer nutritional labelling to mitigate misperceptions and support healthier consumer choices. Further research with larger samples and real‐world settings is recommended to validate these findings.

## Summary

- Organic halo effect on calorie perception: The study shows that foods labelled as organic are perceived as lower in calories when they are high‐calorie.
- Impact on consumption recommendations: The organic label only influences consumption recommendations for low‐calorie foods, which are perceived as less frequently consumable than their conventional counterparts.
- Role of nutritional label reading: Individuals who frequently read nutritional labels are more susceptible to the organic halo effect.

## 1 Introduction

The vast diversity and quantity of available food products require consumers to make numerous decisions about their diet. However, most consumers do not engage in thorough processing of the products they choose, instead relying primarily on heuristics [1] and habits [2]. Consequently, very few consumers appear to scrutinise the products they purchase and consume. In Europe, for example, Grunert et al. [3] observed that only 16.8% of buyers frequently read nutrition information on the food items' labels. It is therefore crucial to comprehend the factors influencing consumer food choices knowing that they do not read the information related to the nutritional value of food. Impression formation associated with food items can be influenced by salient and readily available cues on food packaging, such as labels [4, 5]. They lead to evaluation errors such as a stronger perception of the products' healthiness. Thus, labels such as ‘local’ [6], ‘gluten‐free’ [7] and even ‘vegetarian’ [8] create a halo effect which makes the products perceived as healthier or less caloric.

One of the most studied labels in current research is the organic one. Indeed, numerous studies have observed that organic foods are considered to be lower in calories and fat, but higher in fibre and more nutritious [9]. They are also perceived as equally healthy as nonorganic foods that actually contain significantly less sugar and fat [10]. Furthermore, organic foods are deemed suitable for more frequent consumption [11]. When chosen, they contribute to greater leniency in deviations from a diet or physical activity regimen [11, 12]. This is particularly surprising since the organic label, while providing information about agricultural production practices (Codex Alimentarius, 2007; EC, 2016; USDA, 2016a; IFOAM, 2017a), particularly the absence of synthetic pesticides and fertilisers, does not provide relevant information regarding the nutritional qualities (sugar level, saturated fat level, etc.) and caloric content of foods [13]. Moreover, the nutritional differences among the two are often nonsignificant or highlighted with contradictory results in scientific literacy [14, 15, 16, 17].

### 1.1 The Halo Effect

This phenomenon is commonly known as a halo effect. This cognitive bias occurs when a salient characteristic of an object (in this case, the organic label) influences the evaluation of other dimensions of this item for which no relevant information is available (here the healthiness [18]). The halo effect has been described as a transformation effect [19, 20]: according to the Feature Transformation Framework, the perception of a *target feature*, which is an attribute of an object about which people form assumptions, and which belongs to the *target object* is affected by the *source feature*, which is an attribute that shapes these assumptions and belongs to the *source object*. Notably, these ‘features’ can encompass various dimensions of stimuli, and ‘objects’ can pertain to different types of stimuli (e.g., faces, people, animals, products). When the source feature influences perceptions of the target feature (e.g., food items labelled as vegetarian are perceived as less caloric), feature transformation is said to occur regardless of the effect's direction. A key aspect of this framework is that it does not address the mental processes behind the source's influence on the target. The term feature ‘transformation’ merely describes the effect (i.e., the influence of the source feature on the target feature) without delving into the underlying mental mechanisms driving this effect. This framework thus encompasses diverse well‐known phenomena such as halo and horn effect, evaluative and attribute conditioning or spontaneous trait inference and transference.

One of the most recognised effects of the organic halo in current research is focused on the caloric estimation of foods [8, 21]. Organic‐labelled (source feature) products tend to be perceived as less caloric (target feature) than their noncertified counterparts [21].

### 1.2 Moderators of the Organic Halo Effect

This organic halo effect, while observable in the general population on diverse dependent variables, appears to be moderated by both product‐specific attributes and consumer‐specific characteristics.

At the product level, several research studies have already been conducted. Research indicated that the perceived health and caloric benefits of organic food compared to conventional food are more significant for whole foods than for processed foods [22]. Also, the organic label seems to enhance intention to consume vice food and eliminate the intention to consume virtue food [23]. Virtue and vice products are often described in terms of relative virtues and vices. Relative vices, or ‘wants’, offer immediate enjoyment, such as the rich taste of chocolate cake, but are associated with high‐calorie, long‐term negative effects like weight gain and health risks. On the other hand, relative virtues, also called ‘shoulds’, are less satisfying in the short term but involve fewer negative long‐term consequences and an overall lower calorie content, making them a wiser choice overall (see [24, 25]). On the willingness to pay and actual buying behaviours, UK consumers report being willing to pay 13% more for organic products, and actually pay 9% more compared to conventional products [26]. However, Dutch consumers seem more reluctant to purchase organic in vice than in virtue categories [25]. These initial results seem to show that the magnitude of an organic halo effect may depend on the targeted food, particularly on whether it is perceived as high or low in calories. For example, researchers have found no halo effect for virtue foods such as water [27] or soy milk [28], but have for unhealthy ‐ or vice ‐ foods such as lasagnas [12]. Low‐calorie foods may not be subject to a halo effect for several possible reasons. Here, we propose two. First, since they contain relatively few calories to begin with, there is little room for a perceived caloric reduction driven by the halo effect. Second, high‐calorie organic foods may create a situation of cognitive dissonance, where the contradiction between the ‘healthy’ image of organic products and their high caloric content leads individuals to reassess their perceptions. To resolve this discomfort, they may downplay the calorie content or reframe the product as healthier in other ways, thereby aligning their perception with their expectations [29, 30].

At the individual level as well, several characteristics seem to moderate the organic halo effect. One of the most studied variables is likely pro‐environmental attitudes. Several studies have shown that the halo effect is more pronounced among pro‐environmental persons [11, 22, 31]. Surprisingly, regarding these previous results, people who buy organic food more often seem to be less susceptible to the organic halo effect [9]. The authors suggest that being more familiar with what the organic term entails may encourage deliberative processing and lead to more accurate assessments of the organic label. This aligns with another result from the same experiment showing that participants who frequently read nutrition labels show a weaker organic halo effect. This suggests again that such consumers may engage in more deliberative processing while evaluating organic products, which in turn would reduce caloric estimation errors.

### 1.3 Aim of the Study

Our study aims to experimentally test if the organic halo is more pronounced on high caloric items than on low caloric items, resulting in a lower caloric evaluation and a higher consumption recommendation of organic highly caloric dishes.

Also, we explore interindividual differences on the organic halo: we hypothesise that individuals with a high nutritional information reading habit will be less sensitive to the organic label and make more precise evaluations.

## 2 Methods

### 2.1 Participants and Procedure

One hundred ninety‐eight participants were recruited online through the EasyPanel service in exchange for payment. The study was described to participants as ‘a study aiming to better understand eating habits’. It was conducted in France among adults aged 18 and over encompassing a diverse range of socio‐demographic profiles (see Table 1 for the sample's socio‐demographic details). Participants first provided their informed consent. Then, they were randomly assigned either to the organic or the conventional food group. In each group, 20 items were displayed (10 highly caloric and 10 low‐caloric). Each item was presented alone on a page with its picture, some nutritional information (i.e., kcal, proteins, carbohydrates, and fat), and whether it was organic. Below this, participants were required to evaluate the calories and the recommended frequency of consumption (i.e., how often a food item can be consumed) using Likert scales. Finally, they provided some demographic information.

**Table 1:** Descriptive information of the study sample.

Age	*M* = 47.45 (SD = 14.69)
Gender	
Man	97
Woman	100
Other	1
Nationality	
French	196
Other	2
Number of respondents	198

This study was pre‐registered on AsPredicted before data collection (pre‐registration link: https://aspredicted.org/7vz7-gf75.pdf). The pre‐registration included the study design, hypotheses, planned sample size, exclusion criteria, and planned analyses.

### 2.2 Stimuli and Measures

#### 2.2.1 Stimuli

We selected 10 high‐calorie and 10 low‐calorie food items from the *Centre d'information sur la qualité des aliments* database^1^ (Ciqual), produced by the French Agency for Food, Environmental and Occupational Health Safety (ANSES; see Table 2 for the list). For each, we generated an illustrative image through Dall‐E using the same prompt: ‘Photo taken from above. White plate. White background. *Name of the dish*’.

**Table 2:** Food items characteristics.

Product	Calories per 100 g	Proteins per 100 g	Carbohydrates per 100 g	Fat per 100 g
High calorie				
Four cheese pizza (pizza quatre fromages)	267	13.1	28.2	10.5
Spinach and goat cheese pie (Tarte épinards chèvre)	244	7.08	22.2	13.6
Aligot (potatoes with fresh tomme cheese; aligot, préemballé)	195	8.06	9.5	13.3
Cheese puff pastry or savory pastry (Feuilleté ou Friand au fromage)	302	8.25	28.1	17.1
Cheese risotto (Risotto aux fromages)	194	5.65	30.9	5.16
Cheese omelette (Omelette au fromage)	255	17.7	2.56	19.3
Potato brick (Brick à la pomme de terre)	215	3.25	19.6	13.4
Fried chickpea balls/Falafel (Falafel/boulettes de pois chiche frites)	252	8.25	18.7	14.4
Vegetable cereal patty (Galette de céréales aux légumes)	211	5.31	28	7.6
Onion pie (Tarte à l'oignon)	280	7	23.5	17.2
Low calorie				
Tomato soup (Soupe de tomates)	38.7	0.74	6.27	0.99
Creamed carrot puree (Purée de Carottes cuisinée à la crème)	44.4	1.44	4.57	1.6
Vegetable stir‐fry without mushrooms (Poêlée de légumes sans champignons)	81.2	2.6	12.1	1.77
Vegetable risotto (Risotto aux légumes)	120	2.74	18.4	3.8
Mixed vegetables (Macédoine de légumes)	54.5	3.48	6.8	0.28
Coleslaw (Salade de choux)	105	0.94	5.78	8.1
Vegetable spring rolls (Rouleaux de printemps aux légumes)	103	4.57	16.6	1.82
Vegetable couscous (Couscous de légumes)	108	4.32	16.4	2.25
Baked eggplant (Aubergines au four)	33.8	1.31	3.83	0.3
Vegetable Ravioli in Tomato Sauce, (Raviolis aux légumes, sauce tomates)	91.1	2.88	13.5	2.45

#### 2.2.2 Measures

##### 2.2.2.1 Calories Evaluations

Participants evaluated the calorie content of the food items using a procedure inspired by Schuldt and Schwarz [11]. They were asked to compare the presented dish (organic vs*.* conventional) to other brands producing similar products. Specifically, participants responded to the following question: *‘*Compared to other brands producing this type of [product name], how many calories do you think this dish contains?*’*. Responses were recorded on a 7‐point Likert scale ranging from 1 (‘fewer calories’) to 7 (‘more calories’).

##### 2.2.2.2 Consumption Recommendation

Participants also provided consumption recommendations following a procedure similar to Schuldt and Schwarz [11]. They were asked to evaluate the frequency with which the presented dish (organic vs*.* conventional) could be consumed compared to other brands producing similar products. Specifically, participants answered the following question: *‘*Compared to other brands producing this type of product, how frequently do you think this [product name] can be consumed?*’*. Responses were recorded on a 7‐point Likert scale ranging from 1 (‘less often’) to 7 (‘more often’).

##### 2.2.2.3 Information Research

Participants responded on a 5‐point Likert scale ranging from 1 = never to 5 = always, indicating how often they read the nutritional information on the products they consume.

### 2.3 Data Analysis and Results

First, the low‐calorie items have been recoded as −1 and the high‐calorie items as 1. Since the responses are nested (one participant evaluated 20 food items), we decided to run multilevel regression models using the lme4 package [32]. The significance level for analysis has been set at *α* = 0.05.

We acknowledge that we deviated from the pre‐registered analysis plan. Initially, we planned to use multiple linear regression models to test our hypotheses. However, upon further consideration, mixed‐effects models were deemed more appropriate due to the nested structure of the data. This approach allowed us to account for within‐subject variability and optimise the accuracy of our statistical analyses.

#### 2.3.1 Effect of the Label on Calorie Estimation

In the first model, we included a random intercept for the participant and for the food item, and the label (organic vs. conventional) was added as the predictor of calorie estimation. Unexpectedly, the label had no impact on calorie estimation, *β* = 0.05, 95% CI [−0.18, 0.27], *p* = 0.667.

#### 2.3.2 Moderation of the Caloric Characteristics on the Label Effect on Calorie Estimation

**Figure 1:**
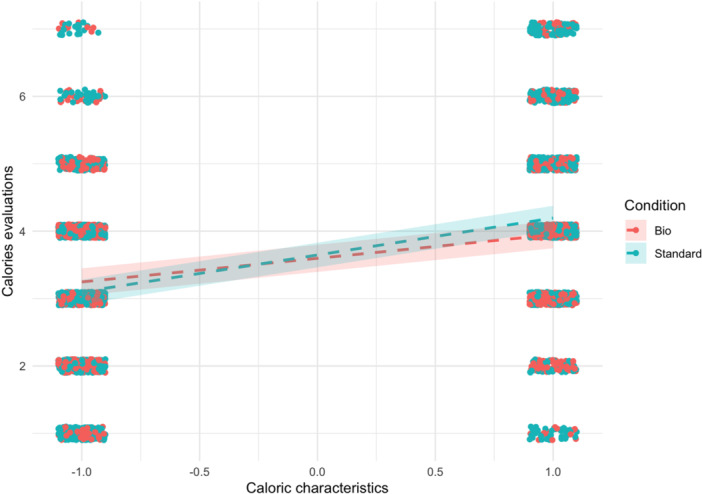
Moderation of the caloric characteristics on the label effect on calorie estimation.

In the second model (see Table 3), we expanded upon the previous model by including the interaction between the label (organic vs. conventional) and the caloric characteristics of the food items (high‐calorie vs. low‐calorie). We identified a significant two‐way interaction, with *β* = 0.20, 95% CI [0.13, 0.26], *p* < 0.001. This suggests that the undermining effect of the organic label is solely observed in high‐calorie items (see Figure 1).

**Table 3:** Moderation of the caloric characteristics on the label effect on calorie estimation.

Predictors	Calories evaluations		
	**Estimates**	**95% CI**	***p***
(Intercept)	3.60	3.41, 3.78	**< 0.001**
CaloricLevel	0.35	0.27, 0.44	**< 0.001**
Condition	0.05	−0.18, 0.27	0.667
Caloric level × condition	0.20	0.13, 0.26	**< 0.001**
Random effects			
σ^2^	1.11		
τ_00 responseId_	0.59		
τ_00 item_	0.02		
ICC	0.35		
*N*_responseId_	198		
*N*_item_	20		
Observations	3960		
Marginal *R* ^2^/conditional *R* ^2^	0.115/0.429		

*Note:* Bold values are statistically significant.

#### 2.3.3 Moderation of the Caloric Characteristics and the Tendency to Search for Nutritional Information on the Label Effect on Calorie Estimation

**Figure 2:**
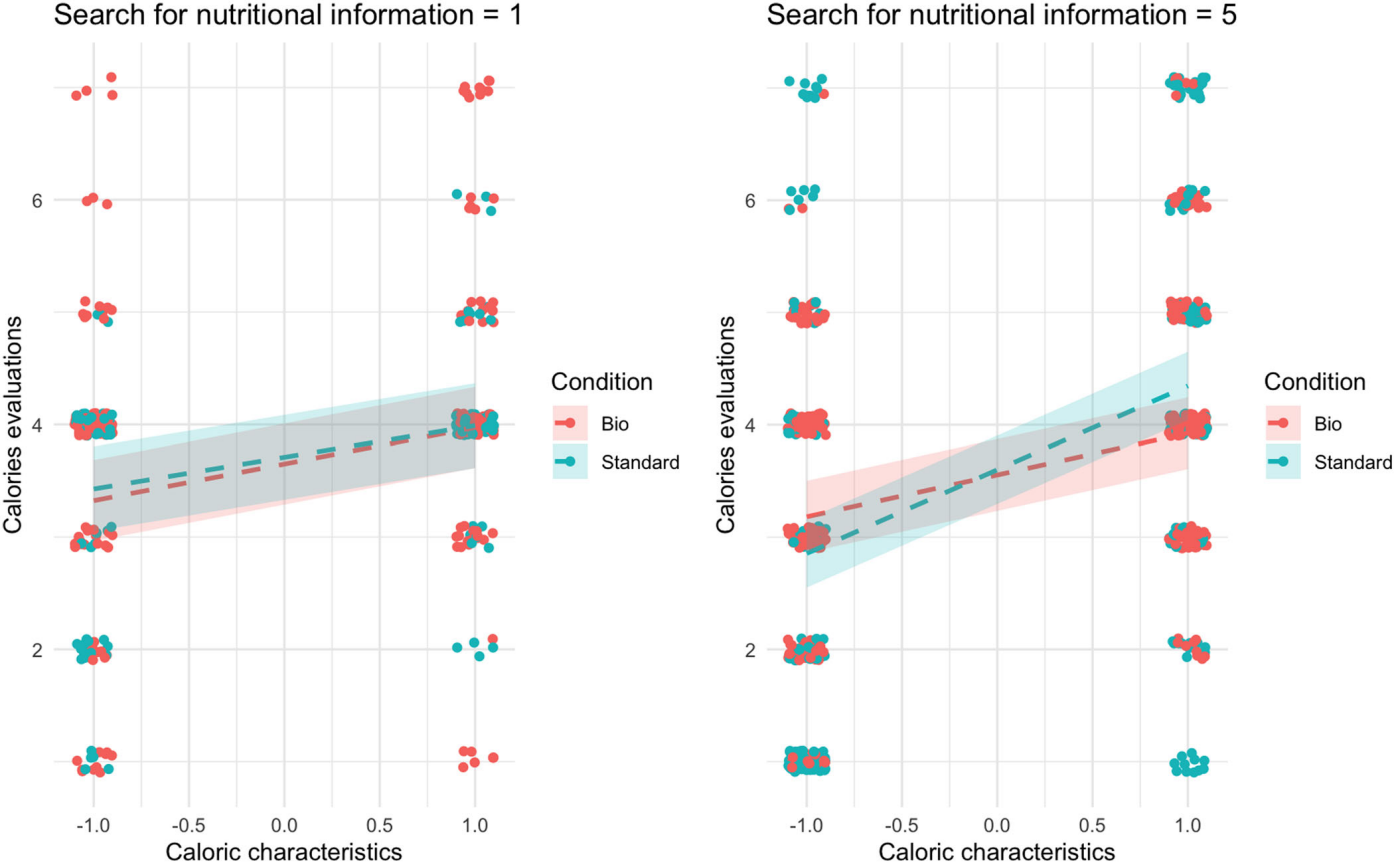
Moderation of the caloric characteristics and the tendency to search for nutritional information on the label effect on calorie estimation.

In the third model (see Table 4), we further extended the previous model to include the three‐way interaction between the label, the caloric characteristics of the food items, and the tendency to search for nutritional information. We discovered a significant three‐way interaction, with *β* = 0.10, 95% CI [0.05, 0.16], *p* < 0.001. This suggests that participants who infrequently seek out nutritional information are unaffected by the label. However, those who habitually look for nutritional information show increased sensitivity to the organic label when evaluating high‐calorie dishes (see Figure 2).

**Table 4:** Moderation of the caloric characteristics and the tendency to search for nutritional information on the label effect on calorie estimation.

Predictors	Calories evaluations		
	Estimates	95% CI	*p*
(Intercept)	3.67	3.22, 4.13	**< 0.001**
Caloric level	0.31	0.16, 0.46	**< 0.001**
Condition	0.06	−0.59, 0.72	0.850
Search info	−0.02	−0.15, 0.11	0.721
Caloric level × condition	−0.15	−0.34, 0.04	0.130
Caloric level × search info	0.01	−0.03, 0.05	0.550
Condition × search info	−0.00	−0.19, 0.19	0.972
(Caloric level × condition) × search info	0.10	0.05, 0.16	**< 0.001**
Random effects			
σ^2^	1.10		
τ_00 responseId_	0.59		
τ_00 item_	0.02		
ICC	0.36		
*N*_responseId_	198		
*N*_item_	20		
Observations	3960		
Marginal *R* ^2^/conditional *R* ^2^	0.120/0.435		

*Note:* Bold values are statistically significant.

#### 2.3.4 Effect of the Label on Frequency Recommendation

In the fourth model, we included a random intercept for the participant and for the food item, and added the label (organic vs. standard) as the predictor of the frequency recommendation. The analysis revealed that the label significantly influenced the frequency recommendation, with *β* = 0.27, 95% CI [0.04, 0.51], *p* = 0.021. This suggests that standard items are recommended to be consumed more frequently than organic ones.

#### 2.3.5 Moderation of the Caloric Characteristics on the Label Effect on Frequency Recommendation

**Figure 3:**
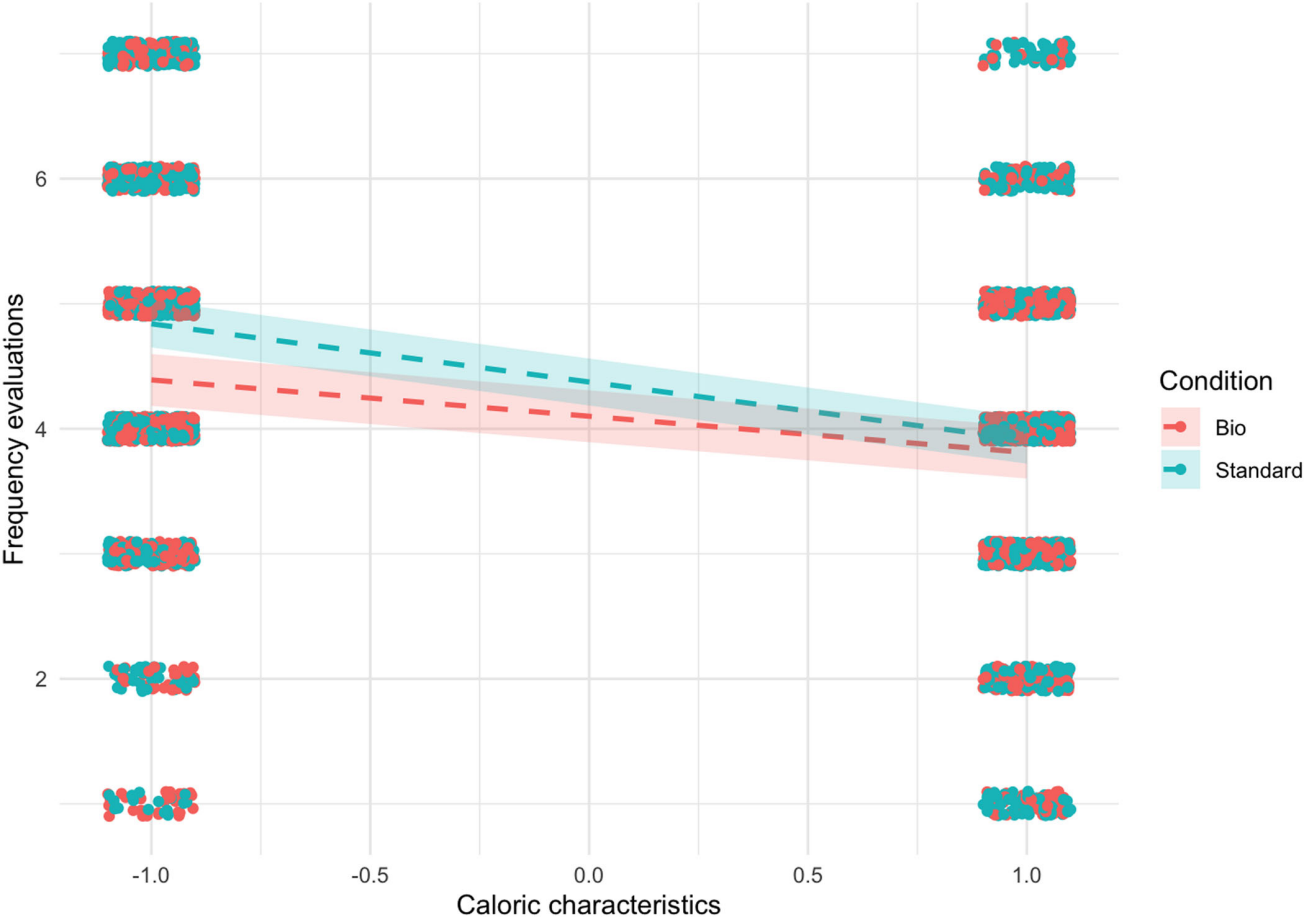
Moderation of the caloric characteristics on the label effect on frequency recommendation.

In the fifth model (see Table 5), we expanded the previous model by including the interaction between the label and the caloric characteristics of the food items. We identified a significant two‐way interaction, *β* = −0.17, 95% CI [−0.24, −0.11], *p* < 0.001, indicating that only the low‐calorie dishes are perceived as more frequently consumable (see Figure 3).

**Table 5:** Moderation of the caloric characteristics on the label effect on frequency recommendation.

Predictors	Frequency recommendation		
	Estimates	95% CI	*p*
(Intercept)	4.10	3.91, 4.29	**< 0.001**
Caloric level	−0.29	−0.38, −0.20	**< 0.001**
Condition	0.27	0.04, 0.51	**0.021**
Caloric level × condition	−0.17	−0.24, −0.11	**< 0.001**
Random effects			
σ^2^	1.18		
τ_00 responseId_	0.63		
τ_00 item_	0.03		
ICC	0.36		
*N*_ResponseId_	198		
*N*_Item_	20		
Observations	3960		
Marginal *R* ^2^/conditional *R* ^2^	0.088/0.413		

*Note:* Bold values are statistically significant.

#### 2.3.6 Moderation of the Caloric Characteristics and the Tendency to Search for Nutritional Information on the Label Effect on Frequency Recommendation

**Figure 4:**
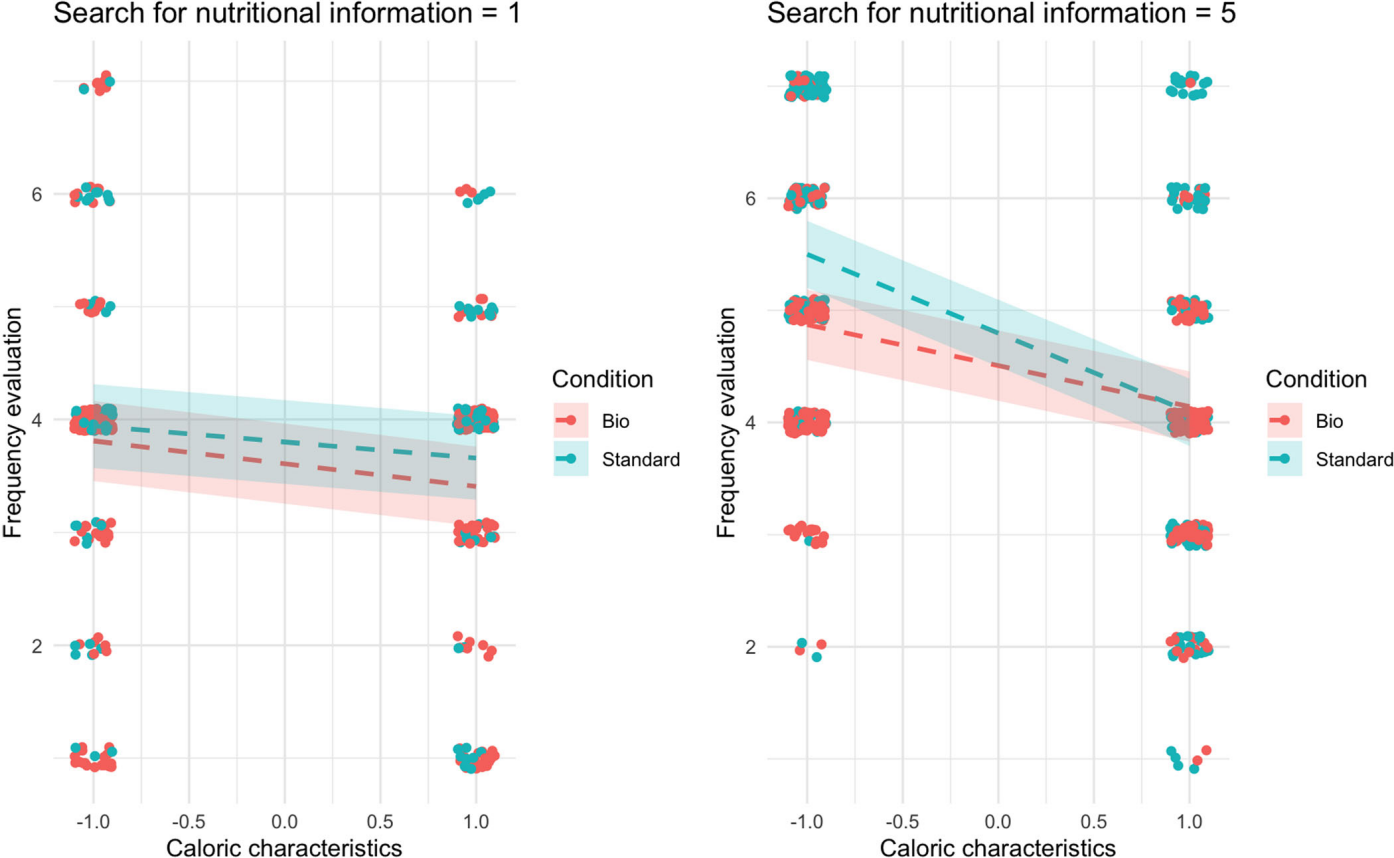
Moderation of the caloric characteristics and the tendency to search for nutritional information on the label effect on frequency recommendation.

In the final model (see Table 6), we augmented the previous model by incorporating the interaction between the label, the caloric characteristics of the food items, and the tendency to search for nutritional information. We observed a significant three‐way interaction, *β* = −0.10, 95% CI [−0.16, −0.04], *p* < 0.001. This suggests that participants who do not frequently seek nutritional information are mostly unaffected by the label. Conversely, those who habitually search for nutritional information are more inclined to recommend standard items over organic ones when the items are low‐calorie (see Figure 4).

**Table 6:** Moderation of the caloric characteristics and the tendency to search for nutritional information on the label effect on frequency recommendation.

Predictors	Frequency recommendation		
	Estimates	95% CI	*p*
(Intercept)	3.39	2.94, 3.83	**< 0.001**
Caloric level	−0.16	−0.31, −0.01	**0.039**
Condition	0.17	−0.47, 0.81	0.609
Search info	0.22	0.10, 0.35	**0.001**
Caloric level × condition	0.16	−0.04, 0.36	0.112
Caloric level × search info	−0.04	−0.08, −0.00	**0.041**
Condition × search info	0.02	−0.16, 0.21	0.795
(Caloric level × condition) × search info	−0.10	−0.16, −0.04	**0.001**
Random effects			
σ^2^	1.17		
τ_00 responseId_	0.56		
τ_00 item_	0.03		
ICC	0.33		
*N*_responseId_	198		
*N*_item_	20		
Observations	3960		
Marginal *R* ^2^/conditional *R* ^2^	0.134/0.422		

*Note:* Bold values are statistically significant.

## 3 Discussion

In this study, we aimed to explore the potential differentiated impact of organic labels on dishes with high and low caloric content. Initially, we found that only dishes with high caloric content and organic labels were underestimated in terms of calories. This indicates that the organic label does not undermine the perception of calories for all foods, regardless of calories, but significantly affects high‐calorie ‐vice‐ ones. This is particularly significant as it may lead individuals to consume more calories than they intend. A possible explanation for this effect is that high‐calorie products inherently have a higher total caloric value, which provides a larger numerical range for potential reductions in perceived calories when influenced by the organic halo effect. In contrast, low‐calorie products, due to their already limited baseline caloric content, offer a much smaller scope for underestimation, making any reduction less substantial both in absolute and perceptual terms. However, the results of our study indicate that low‐calorie organic foods are perceived to have slightly higher calorie content than their conventional counterparts. This effect may be attributed to the influence of the organic label on food evaluations. Specifically, high‐calorie foods are perceived as having lower calorie content, while low‐calorie foods are perceived as having higher calorie content, due to the initial perception that they are not overly charged with calories. A second possible explanation could be that the low‐calorie items are thought to be very poor on calories and nutrients and thus the organic label makes them perceived as a bit more nutritious because it would be perceived as healthier. This should be considered with a previous study that showed that an energy bar was perceived as higher in calories when labelled as fair trade [33]. Thus, the most calorically dense organic foods would be considered less caloric than other foods of the same type, and the least calorically dense ones a bit more caloric. In both cases, this would reflect healthier and more nutritious foods. A replication testing directly the perception of nutrients could test this hypothesis.

However, when examining the recommended frequency of consumption, we observed no difference in high‐calorie products. Organic high‐calorie foods were considered as just as consumable as their nonorganic counterparts in terms of frequency. This suggests that if organic is associated with a higher calorie intake, it is not due to increased consumption frequency but rather to an underestimation of their caloric content, leading to occasional excessive eating. In other words, where one might not typically consume a biscuit, they might allow themselves an organic biscuit, thereby making the difference occasional rather than regular. Conversely, for low‐calorie items, conventional foods are viewed as more frequently consumable, potentially because they are perceived as healthy and less expensive than their organic counterparts. This perception could be particularly relevant in the highly inflationary context of Western Europe. Indeed, in 2022, purchases of organic food products in France decreased by 4.6% (*French Ministry of Agriculture and Food Sovereignty, 2023*). Future studies should aim to replicate our observed results in diverse economic environments to better understand their generalisability.

Finally, we noted that the tendency to seek nutritional information moderates the organic halo effect. The more participants were accustomed to looking for nutritional information, the more they underestimated the caloric content of organic high‐calorie dishes. Our results suggest that these particularly attentive individuals heavily rely on their cognitive biases, thus incorrectly evaluating food products. This contrasts with Lee et al. [9], who observed that frequent nutrition label readers exhibited a weaker organic halo effect. Several methodological differences may account for this discrepancy. First, Lee et al. [9] conducted an in‐person experiment in a shopping mall, where participants physically tasted organic and conventionally labelled products before providing their evaluations. In contrast, our study relied on an online experimental paradigm where participants assessed food items based on images and descriptions rather than direct consumption. Second, an important distinction lies in the number of food items evaluated. Lee et al. [9] asked participants to assess only three food items (i.e., cookies, chips, yogurt), whereas our study included 20 items spanning both high‐ and low‐calorie categories. Finally, Lee et al. [9] used a within‐subjects design, where each participant tasted both an organic‐labelled and a conventionally labelled version of the same food item, allowing direct comparison between the two conditions. In contrast, our study employed a between‐subjects design. These differences may have influenced the cognitive processing of information. Given that discrepancy, new studies must be conducted to replicate and refine our comprehension of this variable. We can hypothesise that individuals who are mindful of their diet, may be more drawn to the organic label. Education, familiarity with environmental and health issues, and gender are linked to a preference for environmental, social, and health attributes [34], and a favourable attitude towards the organic label is associated with a stronger halo effect [11, 22]. In terms of caloric foods, organic items might be considered healthier and thus perceived as containing fewer calories.

Regarding consumption frequency, standard low‐calorie products are perceived as more suitable for regular consumption, but only among individuals who frequently seek nutritional information. No difference was observed for high‐calorie products. This suggests that those most concerned with their health recognise that low‐calorie products offer a health benefit and can thus be consumed more frequently. Moreover, those from standard agriculture could be consumed even more as they cost less to purchase.

We thus observed a significant dissociation between calorie perception and frequency of consumption recommendations. The two do not appear to be perfectly linked, as high‐calorie organic foods are underestimated in terms of caloric content, yet both organic and nonorganic high‐calorie products show no difference in recommended consumption frequency. One possible future hypothesis is that this might lead to occasional overconsumption of calories, without necessarily persisting over time. This could manifest, for example, in an individual consuming an entire pack of cookies at once, under the belief that it constitutes a minor indulgence because the cookies are organic. However, this behaviour would likely remain infrequent.

### 3.1 Limitations

This study, however, has several limitations. It was conducted experimentally and online, which does not allow access to actual consumer behaviours. In Vivo effects could thus be quite different from those observed here. Moreover, our sample included only 198 participants, making it difficult to generalise the results, particularly those concerning individual differences. A larger‐scale replication is essential for future research to reassess both the differential impact of calorie content on the organic halo effect based on the product type (high vs. low calorie) and the moderating role of nutritional information reading habits. Finally, although our dependent variable measures are commonly used and very informative, they could make interpretation more complex as participants' evaluations depend not only on the target product but also on their mental representation of comparable products, which may not be immediately accessible in memory. Another study, using more direct measures (e.g., caloric estimation; possible frequency of consumption per week or month), could provide a clearer assessment by eliminating the need for comparisons.

## 4 Conclusion

This study observed a differentiated effect of the organic label according to type of food. Thus organic foods are perceived to be less caloric when they are vice and more caloric when they are healthy. Moreover, this effect is differentiated according to the habit of reading nutritional information. Individuals who regularly read nutritional information seemed to rely more on the organic label when making evaluations, as we observed a stronger organic halo effect among them. On the perceived frequency to eat variable, on the contrary we observed that standard food is considered as more frequently consumable and this is particularly the case for participants used to reading the nutritional information. We can conclude that individuals are sensitive to diverse influences when making food decisions and that the regulatory must help them by implementing science based interventions such as nutri‐score, a front‐of‐package label that informs consumers about the overall healthfulness of a product and have demonstrated in both laboratory and field settings that it can effectively modify consumer behaviour [35, 36].

## Author Contributions

**Théo Besson:** writing – original draft, methodology, formal analysis, data curation, conceptualisation. **François Durand:** writing – original draft, methodology, formal analysis, data curation, conceptualisation. **Oulmann Zerhouni:** writing – original draft, supervision, methodology, formal analysis, conceptualisation.

## Ethics Statement

This study was conducted in accordance with the ethical principles outlined in the Declaration of Helsinki and complies with applicable French regulations on research involving human subjects (Loi Jardé). Formal ethical approval was not required for this study. However, informed consent was obtained from all participants, and the study adhered to ethical guidelines to ensure their privacy and well‐being.

## Conflicts of Interest

The authors declare no conflicts of interest.

## Data Availability

The data supporting the findings of this study will be made available on the Open Science Framework (OSF) at https://osf.io/nz2yq/?view_only=96871e64f40b4ab68d395ba07f0527c3 upon publication.

## Endnotes

1 Food Quality Information Centre database in English.
